# Identification of *Arvicola terrestris scherman* Sperm Antigens for Immune Contraceptive Purposes

**DOI:** 10.3390/ijms22189965

**Published:** 2021-09-15

**Authors:** Areski Chorfa, Chantal Goubely, Joelle Henry-Berger, Rachel Guiton, Joël R. Drevet, Fabrice Saez

**Affiliations:** UMR GReD Institute (Génétique Reproduction & Développement) CNRS 6293, INSERM U1103, Equipe «Mécanismes de L’infertilité Mâle Post-Testiculaire», Université Clermont Auvergne, 63000 Clermont-Ferrand, France; areski.chorfa@uca.fr (A.C.); chantal.goubely@uca.fr (C.G.); joelle.henry@uca.fr (J.H.-B.); rachel.guiton@uca.fr (R.G.)

**Keywords:** sperm cells, contraceptive vaccine, wild rodent

## Abstract

The cyclical proliferation of the wild fossorial rodent *Arvicola terrestris scherman* (*ATS*) is critical in mid-mountain ecosystems of several European countries. Our goal is to develop an immunocontraceptive vaccine to control their fertility, as a sustainable alternative to chemical poisons currently used. Indeed, these chemicals cause the death of *ATS* predators and animals sharing their ecosystem, and current laws progressively limit their use, making the development of a targeted vaccination strategy an interesting and efficient alternative. In order to identify species-specific sperm antigens, male and female *ATS* received subcutaneous injections of whole *ATS* spermatozoa to elicit an immune response. The analysis of the immune sera led to the identification of 120 immunogenic proteins of sperm cells. Of these, 15 were strictly sperm-specific and located in different regions of the male gamete. Some of these antigens are proteins involved in molecular events essential to the reproductive process, such as sperm–egg interaction, acrosomal reaction, or sperm motility. This approach not only identified a panel of immunogenic proteins from *ATS* sperm cells, but also demonstrated that some of these proteins trigger an immune response in both male and female *ATS*. These spermatic antigens are good candidates for the development of a contraceptive vaccine.

## 1. Introduction

The terrestrial vole, also known as *Arvicola terrestris Scherman* (*ATS)*, is a fossorial rodent living in underground tunnels in mid-mountain regions generally located between 400 and 1500 m above sea level [1]. The proliferation of this rodent shows cyclical variations over a period of about 5 to 6 years, with densities ranging from 50–100 animals/hectare to more than 1000 during outbreaks [2,3]. The excessive cyclical proliferation and expansion of *ATS* colonization areas, since the 1970s, mainly in mid-mountain agronomic and tourist ecosystems, is an increasingly important problem with strong environmental and economic impacts [4,5,6]. At high densities, *ATS* affects the botanical composition of grasslands, causing the regression of legumes and the increase of poor-quality grass and undesirable plants. On cultivated plots, *ATS* can damage cereal crops, orchards, vegetable gardens, vineyards, forests, and ornamental gardens [7,8]. One consequence of *ATS* activity on grassland areas is the presence of mounds that facilitate the ingestion of soil by grazing animals. This leads to an alteration of milk quality characterized by a reduction in protein content and contamination by butyric bacteria. *ATS* outbreaks are also a public health risk because they are vectors of zoonosis. There is a strong relationship between *ATS* density and the risk factor for alveolar echinococcosis [9]. Human alveolar echinococcosis is a serious parasitic disease caused by the larva of a flatworm, *Echinococcus multilocularis*, mainly characterized by hepatic development in a tumorous form, and with no treatment currently available.

Current strategies to control *ATS* populations are dominated by lethal procedures, such as physical trapping and chemical poisoning with baits (wheat, carrot) soaked with the anticoagulant bromadiolone [10]. Although these approaches are effective, they are neither economically viable (trapping) nor environmentally acceptable anymore (chemical control). Indeed, the non-specific nature of chemical control means that collateral damage to off-target fauna (animals with similar diets or predators of *ATS*, such as wild boars, foxes, ermines, weasels, birds of prey, and even cats and dogs) is significant [11,12]. Moreover, although chemical control is performed in a reasoned manner (monitoring of outbreaks, treatment under administrative order, controlled distribution of baits, abandonment of treatments in periods or areas of high densities, etc.) the contamination of ecosystems (groundwater, rivers, sewage treatment plants, etc.) by bromadiolone is a new health risk for the public and wildlife [13]. Recently, in France, the National Agency for Food, Environmental, and Occupational Health and Safety (ANSES) stated that the use of this chemical does not exclude “an unacceptable risk to aquatic organisms, terrestrial vertebrates and groundwater contamination” (AMM no 9800526). This situation urges the development of alternative strategies to fight against the outbreaks of this harmful rodent.

In this context, immunocontraception is an interesting option to limit the reproduction of *ATS*. Immunocontraception involves the administration of a vaccine that induces an adaptive immune response, resulting in subfertility or sterility. Contraceptive vaccines have been used and proven effective in many situations to control the fertility of captive and/or semi-captive wild or domestic animal populations, including horses [14], deer [15,16], squirrels [17], marsupials [18], and African elephants [19]. Briefly, three strategies can be used to achieve vaccine contraception: a post-fertilization strategy by interfering with fetal implantation via a GnRH vaccine (gonadotrophin-releasing hormone) and two pre-fertilization strategies that aim to limit gametic interaction by interfering either with the female gamete or with the male gamete. Anti-GnRH and anti-ZP3 vaccines, with ZP3 being one of the zona pellucida proteins surrounding the oocyte in mammals, are effective in inducing immune contraception [20,21]. These two vaccine approaches, however, present a major pitfall in the context of our interest, which is that they do not allow species specificity given the good sequence conservation of GnRH and ZP3 in mammals. Only the vaccine strategy directed against spermatic antigens makes it possible to envisage a high level of species specificity due to the large number of potential spermatic antigenic targets and the inter-species sequence polymorphism [22,23,24].

While it is rather generally intuitive that sperm antigens can be recognized as non-self by the immune system of female animals, theoretically they should also cause the production of anti-sperm antibodies in males. Indeed, spermatozoa are produced at puberty, long after the repertoire of the self has been established. They are thus considered as non-self even for the male who produces them. The fact that sperm antigens are good triggers of immune responses has been clearly demonstrated by the observation that 70% of men who have undergone a vasectomy develop anti-sperm antibodies or ASA [25,26,27], which often compromises the restoration of fertility in the case of a vaso-vasostomy. These ASA result from the presentation of sperm antigens to the host immune system due to the increased permeability of the blood–testis–epidididymal barriers, a consequence of vasectomy [28]. In addition, ASA have been identified in some human infertile cases [29,30], where they were shown to cause infertility by inhibiting spermatozoa/oocyte binding, reducing sperm motility, reducing sperm penetration into cervical mucus, and altering capacitation and/or acrosomal reaction. 

In this context, immunocontraception studies carried out to date, on different models, used either whole spermatozoa or specific individual sperm antigens. Injection of whole spermatozoa as immunogens was performed in tammar wallabies [31], mice [32], rabbits [33,34], and dogs [35]. However, in these studies, the determination of the sperm antigens triggering the immune response was not conducted, only antibody production and the effects on fertility were described. In rodent models, several sperm antigens (proteins and/or peptides) were studied separately as potential targets for immunocontraception. Even though efficient immune responses and decreased fertility were obtained when using FA1 [36], CRISP1 [37], SPAG9 [38], or tNASP [39], no further studies were conducted, to our knowledge, to control the population expansion of wild (or domestic) species with these immunogens. 

In the present work, we used a whole spermatozoa immunization strategy to identify specific *ATS* sperm antigens for the future development of a contraceptive vaccine.

## 2. Results

### 2.1. Immunoglobulin Detection and Western Blot

**Figure 1:**
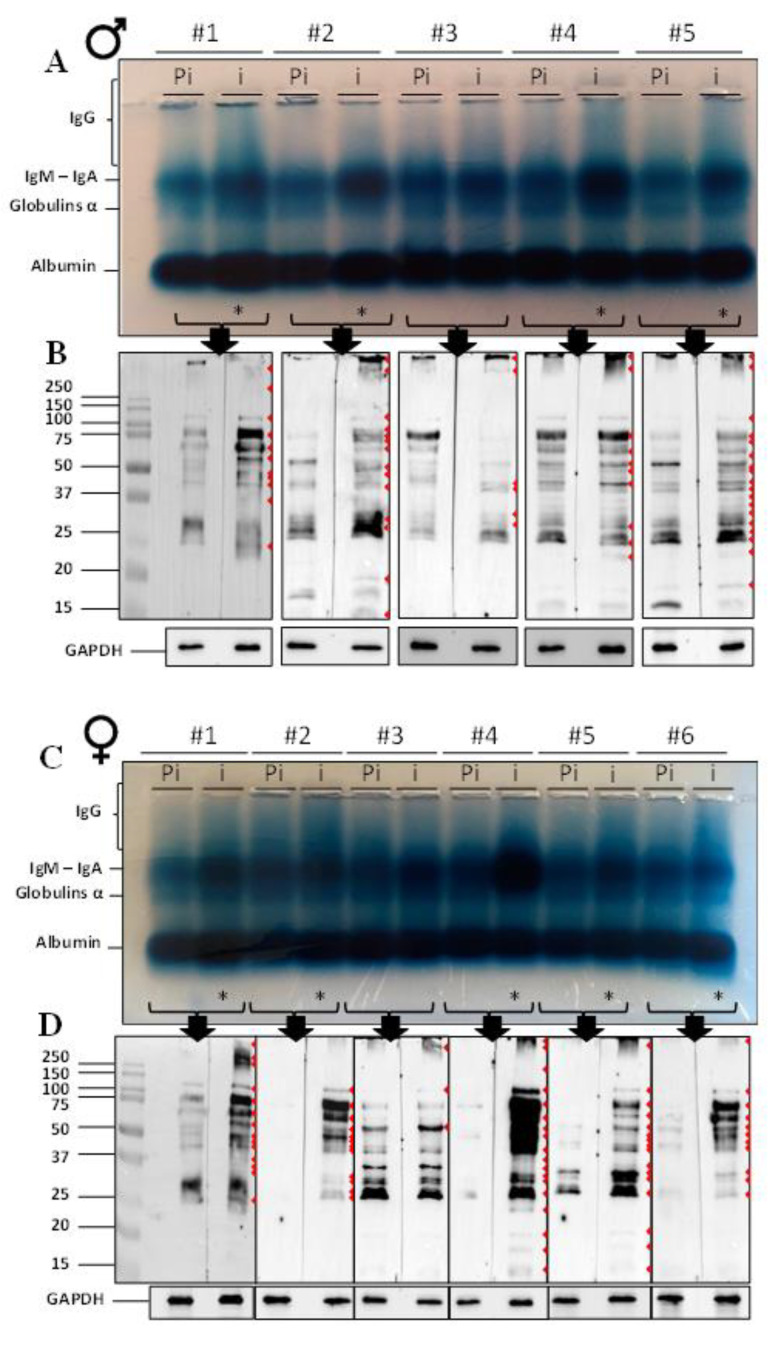
Characterization of serum antibody production after immunization in male and female *ATS*. (**A,C**) Amido black staining. Albumin, alpha globulins, IgM-IgA and IgG profiles in the pre-immune (Pi) and immune sera (i) of five male and six female *ATS*, respectively, were detected in an agarose gel stained with amido black. The stars (*) indicate a strong increase of globulin levels after immunization. (**B,D**) Western blots against *ATS* sperm proteins. Thirty μg of soluble sperm (spz) proteins from *ATS* were loaded on 12% SDS-PAGE gels. The pre-immune (Pi) and immune (i) sera from five male and six female *ATS,* respectively, were used as primary antibodies. The red arrowheads indicate bands differing between pre-immune and immune sera (presence and/or intensity) of the same individual. An anti-GAPDH antibody was used to indicate the similar total protein load in each well (lower panels). Each blot was performed at least twice and each serum was used to probe the spz proteins extracted from at least two different animals.

Amido black staining was used to reveal the different immunoglobulins present in the pre-immune and immune sera of male and female voles after subcutaneous immunization with whole spermatozoa (Figure 1). Comparison of the pre-immune (Pi) and immune (i) sera for each immunized animal showed a clear increase in the corresponding IgM-IgA bands for four males (1, 2, 4, and 5; Figure 1A) and five females (1 to 5; Figure 1C). The IgG appeared as a smear (pattern that extended from the loading slot to the IgM band) that increased in immune sera, compared to pre-immune sera, especially for males 1, 2, 4, and 5 and for females 1, 2 and 4 to 6 (* in Figure 1A,C). 

To verify that anti-sperm IgG were produced in response to immunizations, we examined each serum (pre-immune and immune, Pi and i, respectively) by Western blotting against proteins extracted from non-immunized *ATS* spermatozoa (Figure 1B,D). The detection of anti-sperm antibodies in voles was made possible by the generation of a secondary *ATS*-specific anti-IgG antibody conjugated to HRP (Figure S1). As can be seen in Figure 1B,D, several discrete bands were detected (red arrowheads). In most samples, whether male or female, a greater number of bands and higher intensity bands were detected in immune sera compared to pre-immune sera. This was particularly true for females and less true for males. It was also clear that female immune sera have a much higher reactivity than male immune sera when comparing Figure 1B with Figure 1D. Few animals appeared to be unreactive to immunizations. This was the case for male #3 and female #3, for which there were no obvious differences when comparing pre-immune and immune sera. 

**Figure 2:**
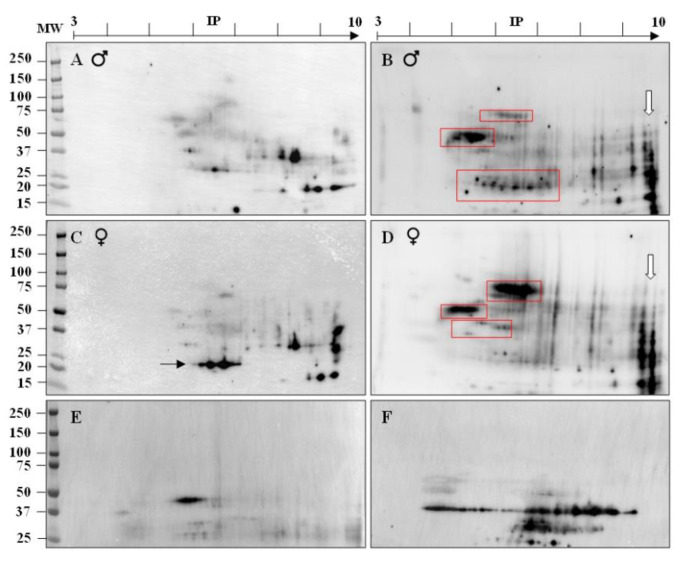
Two-dimensional characterization of *ATS* sperm proteins recognized by ASA. Western blots showing the reactivity of pre-immune (**A**,**C**) and immune (**B**,**D**) sera (pools of sera from 5 male and 6 female *ATS*, respectively) against *ATS* sperm proteins separated by two-dimensional electrophoresis. A black arrow indicates a small group of 20 kDa spots specifically present in female pre-immune sera (**C**), although in both cases some antibodies recognized *ATS* spz proteins (**A**,**C**). The red boxes indicate three groups of spots specifically detected by male immune sera (**B**) and three groups of spots specifically detected by female immune sera (**D**). The white arrows (**B**,**D**) show two groups of very basic spots (IP from 9 to 10) and MW ranging from 12 to 60 kDa detected when blotting with either male or female sera. Positive controls showing blots against the beta-actin (**E**) and GAPDH (**F**) proteins attest the good migration of total extracts of *ATS* sperm proteins in our conditions. Each blot was performed at least twice and each serum was used to probe the spz proteins of at least two different animals.

For further identification of the *ATS* sperm antigens that provoked the immune responses and the generation of ASA, we pooled the sera of the responding animals. The same was done with pre-immune sera. Two-dimensional electrophoresis (2D) was used to analyze pooled pre-immune and immune sera from male and female *ATS* (Figure 2). As revealed by 1D analysis (Figure 1), pre-immune sera contained antibodies directed against sperm proteins (Figure 2A,C). The immunization triggered a higher reactivity for *ATS* sperm proteins in both males and females (blots with immune sera in Figure 2B,D). Among the complex patterns of revealed proteins, red boxed areas on the 2D gels point out protein spots with a certain level of sex specificity, thus showing that males and females put up a different immune response when immunized against whole *ATS* spermatozoa.

### 2.2. ASA Do Target ATS Sperm Surface Proteins

**Figure 3:**
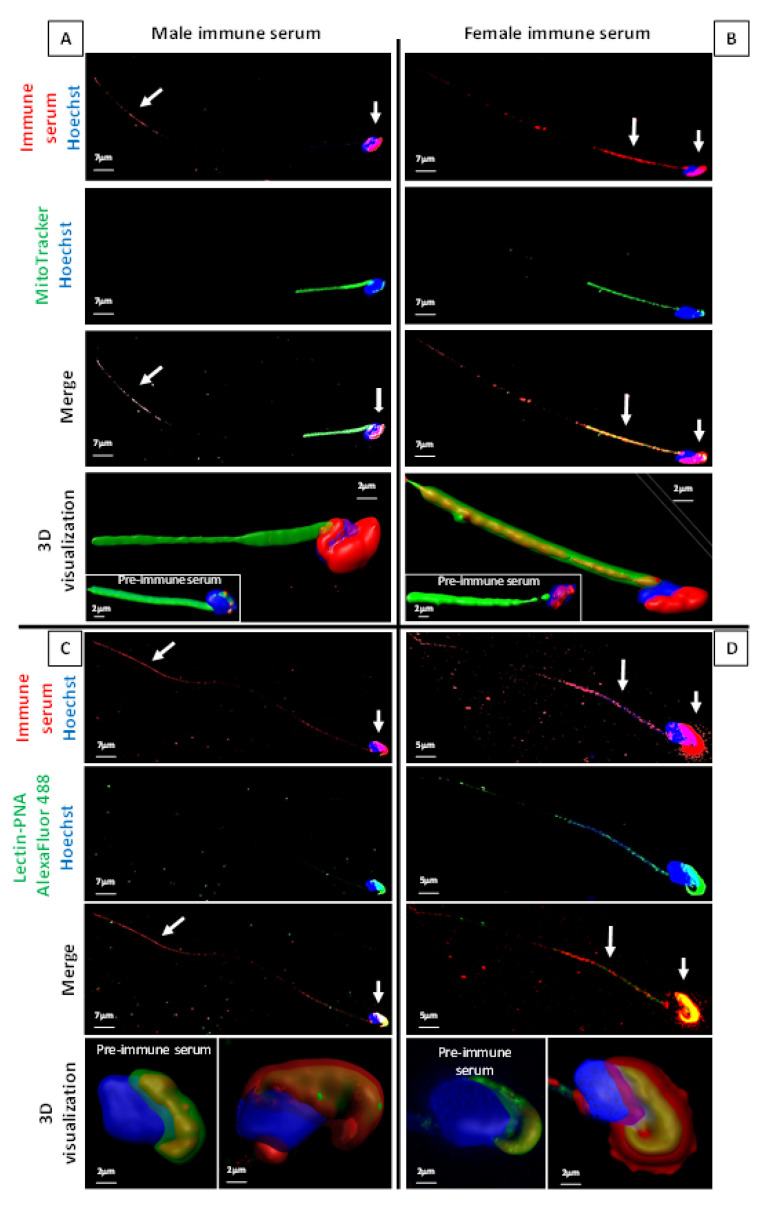
Cellular localization of the proteins recognized by ASA from male and female sera. (**A**,**B**) Fresh *ATS* spermatozoa were triple labelled with the pooled male and female *ATS* immune sera (red), the Mitotracker probe (green), and Hoechst 33342 (blue). Confocal images were processed using Imaris software to obtain a 3D reconstruction of the spermatozoa (lower panels). A focus was made on the middle part of the flagellum and the sperm head. The green staining was made 50% transparent to determine the cellular location of the red label. The white arrows indicate the red labelling of interest, i.e., antigens recognized by ASA. Male immune sera recognized proteins on the surface of the flagellum tip (final piece), a small part of the principal piece, the base of the flagellum (centriole), the head, the acrosome, and very weakly on the intermediate piece, whereas ASA contained in the pool of female immune sera recognized proteins of the intermediate piece of the flagellum, the flagellum base (centriole), the acrosome, and weakly on the flagellum tip and the principal piece. Images obtained with male and female pre-immune sera were processed the same way (insets in the 3D visualization images) and showed weak staining on the acrosome for both male and female, slightly more intense with female pre-immune sera (in accordance with the Western blots and 2D electrophoresis shown in Figure 1 and Figure 2). (**C**,**D**) Fresh *ATS* spermatozoa were triple labelled with the immune serum pool of the male and female *ATS* (red), the lectin-PNA-Alexa Fluor 488 Conjugated (green), and the Hoechst 33342 (blue). The green marker in the pre-immune serum inset and the red marker in the immune serum pictures were made 50% transparent to determine the external cell location of the markers. A focus was made on the acrosome. White arrows indicate the red labelling of interest (antigens recognized by the ASA). The pictures shown in this figure are representative of five experiments performed under these conditions. We used the same pre-immune and immune sera as for Figure 1 and Figure 2. Each pool of sera was tested at least three times to stain spz from at least two different non-immunized animals.

To visualize which regions of the *ATS* spermatozoa carry the antigens that provoked the generation of ASA, we used immunocytochemistry and confocal microscopy (Figure 3). Both pooled immune sera from males and pooled immune sera from females were tested and are presented, respectively, in Figure 3A–C and Figure 3B–D. To better localize the different spermatozoa compartments, a Mitotracker probe (Figure 3A,B) and an Alexa Fluor 488 lectin-PNA probe (Figure 3C,D) were used to, respectively, show the spermatozoa mitochondria-containing midpiece and the sperm head acrosome region. Merged images are provided as well as 3D reconstructions, as allowed by the confocal microscopy technique used. It is interesting to point out that the male immune serum mainly recognized antigens associated with the sperm head and neck (junction flagellum-head) regions, as well as with the terminal part of the flagellum (Figure 3A). In addition to the sperm cell parts recognized by the male immune serum, the female immune serum also distinctly recognized antigens associated with the sperm midpiece, clearly co-localizing with mitochondria. The confocal 3D visualization of sperm head images (lower panels of Figure 3C,D) merging the acrosome lectin-PNA detection and the *ATS* immune sera detection suggested that both male and female immune sera showed reactivity with antigens located in the acrosomal domain and with antigens most likely located in the sperm plasma membrane. 

### 2.3. Identification of Immunogenic ATS Sperm Proteins by Proteomic Analysis

To identify the *ATS* sperm antigens recognized by the ASA, we proceeded with an immunoprecipitation strategy (Figure S2) coupled with quantitative mass spectrometry analysis. As the *ATS* genomic sequence was not yet available, we used the phylogenetically closest genome present in databases, which was that of the *Microtus ochrogaster* (Prairie vole). Overall, 114 different protein targets were identified as differentially pulled down when the immune serum was compared to the pre-immune serum (Table S1a,b). For six of these proteins, the identified peptides matched with a somatic protein and a sperm-specific isoform. The six isoforms were added to the list (Table S2), raising the total number to 120. 

The 120 proteins were classified following several criteria. Table 1 presents the proteins involved in essential steps of the fertilization process, including sperm–egg interaction, zona pellucida penetration, sperm capacitation, and acrosomal reaction. A fifth group, entitled “sperm parts”, contains proteins located in specific subcellular compartments of the spermatozoa, including the nucleus and terminal, and the main and intermediate segments of the flagellum. The proteins involved in metabolic energy processes, located in the mitochondria and chaperones, are listed in the last three groups of this table. As already shown by the immunolocalization of ASA targets on *ATS* spermatozoa (Figure 3), mitochondrial proteins were much more targeted by ASA generated in females than in males. A certain number of proteins could be gathered in three particular groups, as presented in Table 2. Ten proteins belong to a family called moonlighting proteins, which are known for bearing peculiarities in the reproductive function (see the discussion section). Fourteen proteins could be related to phenotypes specific to sexual organs when their gene expression is invalidated, and among those 11 are strictly present in spermatozoa (* in Table 2). Finally, 29 proteins have been previously reported as being part of the epididymosomes, exosomes secreted by the epididymal epithelium and involved in the spermatozoa post-testicular maturation process. Some of the identified proteins have functions related to housekeeping processes and/or non-reproductive functions (Table 3) that could be subclassified into DNA/RNA binding, complement activation, cytoskeletal proteins, lipid metabolism, and a final category called “others”, which includes proteins whose function is not included elsewhere. 

**Figure 4:**
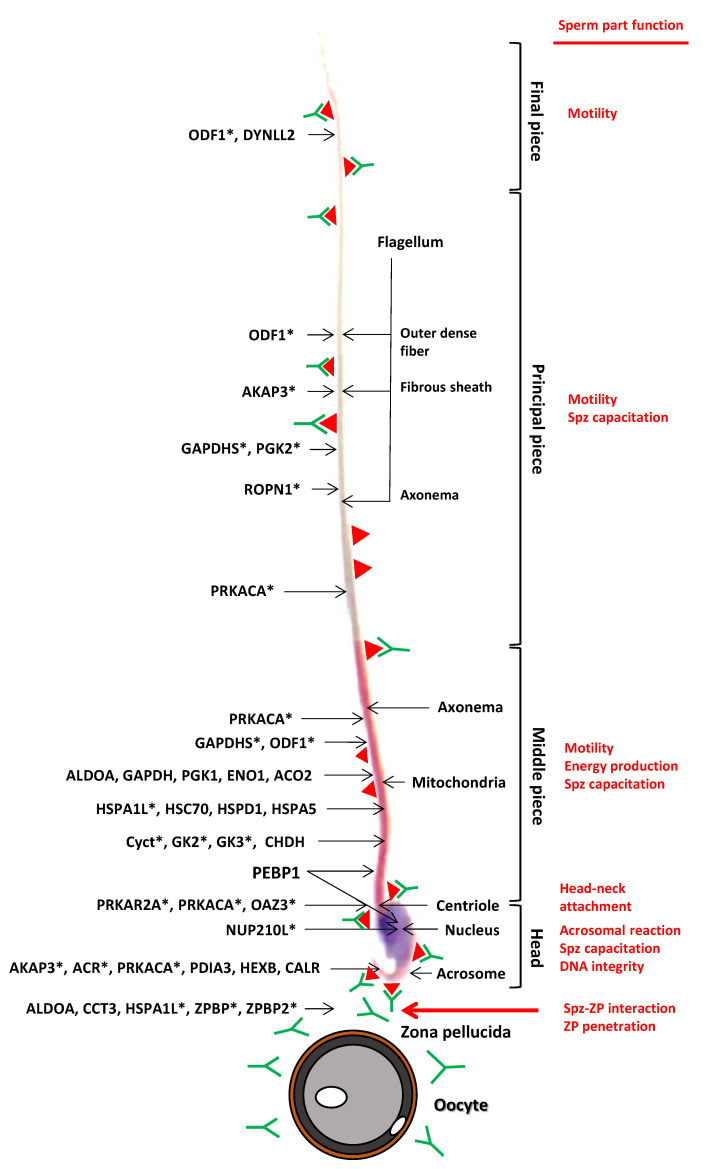
Schematic summary representation of the location of sperm antigens targeted by ASA. Male and female *ATS* immunized with whole spermatozoa of the same species (*ATS*) produced serum ASA (immunoglobulin G- and M-isotypes). This figure represents the interaction between an *ATS* sperm and an oocyte. Some of the antigens identified by LC-MS/MS are represented on the spermatozoon. The red triangles represent proteins targeted by ASA and the green “Y” represent ASA. The stars (*) indicate proteins strictly expressed in the spermatozoa (see Table 1 and Table 2 for more precise protein classification). The sperm parts and functions concerned by the ASA are mentioned on the right part of the figure.

We then arbitrarily set a threshold for the mean fold change at 2 (mFC, corresponding to the quantitative difference for a given protein after its immunoprecipitation with the pre-immune and the immune sera). Among the 120 proteins, 38 had a mFC >2 after IP with both the male and female immune sera (Table S2, not highlighted), 49 were specific to the female sera (Table S2, highlighted in brown), and only four proteins were specific to the male immune sera (Table S2, highlighted in orange). Twenty-nine proteins showed a mFC <2 for both male and female sera (Table S2, highlighted in red). Figure 4 presents the locations of some of the 38 antigens identified with both male and female sera, or sperm-specific proteins, with respect to their cell parts and their known functions as deduced from the literature. This figure illustrates that proteins from all cell parts of the *ATS* spermatozoa triggered an elevated immune response in males and females, in accordance with the IF results presented in Figure 3. Representative of the stronger immune response towards spermatic antigens in female *ATS*, as proposed in the analysis of Figure 1B,D, is the observation that the mean mFC was 8.33 with the female immune sera while it was only 2.51 with the male immune sera. The range of molecular weights and isoelectric points of the identified proteins were in agreement with the two-dimensional electrophoresis analysis in Figure 2B,D. Among the 120 identified proteins, 15 were restricted to the sperm cell lineage (identified by an asterisk in Table S2). 

As we performed four replicates of the LC-MS/MS analysis, we statistically analyzed the 120 identified proteins in a more stringent manner using a *p*-value < 0.05 for the mFC (modelled after a statistical test from Mann and Whitney). It turned out that only 22/120 proteins had a statistically significant fold change (Table S2): six with only the male sera (CHDH, PDIA4, GPI1, TUBB4B, C1QB and RAB2A), 14 with only the female sera (EPHX1, DIABLO, CCT3, HSPA1L, AKAP3, CRAT, ROPN1, HSPD1, PEBP1, GAPDH, GAPDHS, PGK1, PGK2 and DSP), and two with both sera (NUP210L and CAR1). Among those, RAB2A and DSP were significantly detected in lower amounts after immunization, so they did not present any interest for the purpose of this study. It is important to point out that 13 proteins had a *p*-value of 0.057 and were increased after immunization, thus making them potentially interesting. 

The 22 proteins showing a significant difference were then analyzed using the STRING (https://string-db.org (accessed on 7 July 2020)) database with *Mus musculus* as a reference as no closer species was available. The GO analysis for “cellular components” presented in Table 4 showed that the three first categories, when classified by the “strength” parameter, were “sperm fibrous sheath”, “zona pellucida receptor complex”, and “sperm principal piece”.

Altogether, these results demonstrated that an efficient immune response was triggered by immunization of *ATS* with whole spermatozoa, that the different immunogenic proteins are located all along the sperm cell, and some of them are strictly sperm-specific (GAPDHS, HSPA1L, and AKAP3).

### 2.4. Immunization of Male ATS with Peptides from Two Candidate Proteins

**Figure 5:**
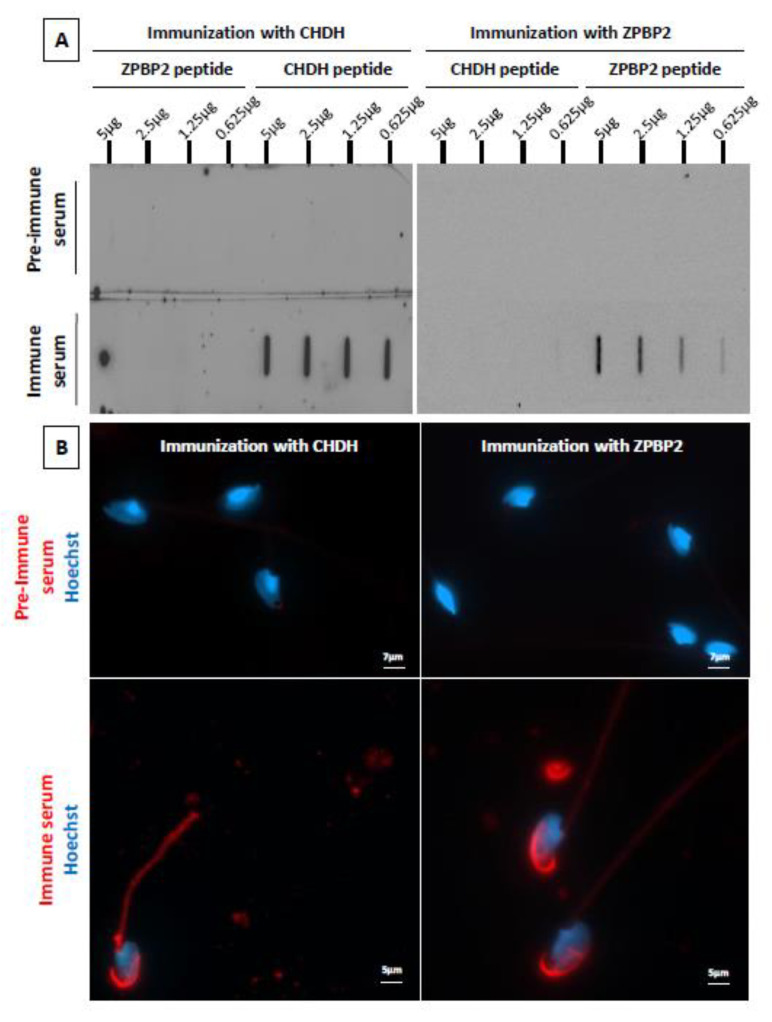
Immune response of male *ATS* immunized with species-specific peptides. Male *ATS* were immunized with a peptide of the CHDH or the ZPBP2 protein. (**A**) The presence of IgG in the pre-immune and immune sera was detected by a slot blot against decreasing quantities of the injected peptide. The specificity of the response was evaluated by blotting each serum against the two peptides. (**B**) Fresh *ATS* spermatozoa were double labelled with male *ATS* pre-immune or immune sera (red) and Hoechst 33342 (blue) and observed using an epifluorescence microscope (Zeiss) equipped with a digital camera, and the micrographs were taken using Zen software (Zeiss). The results in (**A**,**B**) are representative of experiments obtained with three different immunized male *ATS* for each peptide.

The presence of serum IgG specifically directed against the injected peptides was clearly visible using slot blot detection (Figure 5A). The intensity of the immune response was higher for CHDH compared to ZPBP2, as shown by the high signal intensities obtained for CHDH, even with the lowest amount of peptide deposited. This is in accordance with the results obtained with the proteomic analysis (see Table S2). After immunization with the CHDH peptide, the immune sera detected a protein present on the acrosome and in the midpiece (Figure 5B, left panel). CHDH is a mitochondrial protein, the staining of the midpiece was thus expected. More surprisingly, an intense staining was also revealed on the acrosome with the immune sera, a point that will be discussed. Immunization against ZPBP2 triggered the production of IgG binding to a protein mainly present on the acrosome (Figure 5B, right panel), in accordance with what was expected. 

These two immunization experiments with *ATS*-specific peptides for CHDH and ZPBP2 confirm the high potential of the identified proteins for the development of an immunocontraceptive vaccine. 

## 3. Discussion

The objective of this study was to identify antigenic and immunogenic targets on *ATS* spermatozoa that could form the basis of a contraceptive vaccine. To this end, male and female *ATS* were immunized with whole spermatozoa and their sera were evaluated for the presence of anti-sperm antibodies. The sera were then used to identify the sperm antigens that triggered the immune responses using a combination of 2D gel electrophoresis, immunocytochemistry, immunoprecipitation, and LC-MS/MS mass spectrometry. To our knowledge, to date, five studies reporting immunizations using whole sperm have been performed in different animal models, such as tammar wallabies [31], mice [32,40,41], opossum brushtails [18], and dogs [35]. In these studies, only qualitative analyses of triggered immune responses and consequences on fertility were reported. No identification of the antigens targeted by the generated ASA was performed. This study revealed that 120 different sperm proteins had the capacity to trigger an immune reaction in *ATS* under our experimental conditions. Our strategy allowed the identification of the totality of these proteins, thus providing a very interesting source of putative antigens to use in a future contraceptive vaccine. 

Not surprisingly, *ATS* females showed a higher immune response to sperm antigens than *ATS* males, as confirmed by the higher number of proteins showing a significant difference after IP and LC-MS/MS analysis (14 vs. 6 for the males, with two proteins for both sexes). The overall mFC for proteins identified with female sera was also higher than for male sera (8.33 vs. 2.51, respectively). This was expected as spermatic antigens are definitely foreign to the female immune library, while for the male, a tolerogenic status is established with respect to some spermatic antigens to avoid self-reactivity [42]. This may partly explain why male *ATS* do not react strongly to all autoantigens. In addition, it is well documented that there are sex-related differences in the ability to respond to vaccinations. In humans, women have been found to produce more antibodies and have higher B-cell counts than men [43,44]. This phenomenon has been more recently associated with the observation that estradiol, at physiological concentrations, stimulates antibody production [40].

In addition to this quantitative aspect, we noted that the immune responses of female *ATS* were, in part, qualitatively distinct from those of male *ATS*. Although both male and female animals reacted to sperm antigens located on the sperm head and the acrosomal region, it was clear that part of the immune response of female *ATS* to spermatozoa was specific to antigens located in the midpiece, mainly of mitochondrial origin. In contrast, male *ATS* showed no reactivity against sperm antigens located in the midpiece, but showed specific immune responses against antigens located at the flagellum tip (terminal end). Female *ATS* immunization triggered intense immune responses against antigens from sperm mitochondria, a fact that is not in accordance with the maternal origin of mitochondria inheritance. A possible cause would be that proteins of the male germline mitochondria are antigenically distinct or present at different levels. Such a mitochondrial sexual dimorphism has already been suggested [45,46]. Despite the fact that these sperm-mitochondria-associated antigens triggered relatively good immune responses in female *ATS,* they are unlikely to be good candidates for a species-specific vaccine. Indeed, these antigens are mainly proteins involved in energy metabolism, a critical function for sperm motility [47], but without any cell and/or species specificity [48]. Apart from the special case of mitochondrial sperm antigens, it is surprising to note that only male *ATS* built immune responses against antigens located at the flagellum tip, while female *ATS* did not. Once again, this confirms the idea that there are sex-specific immune responses and, within each sex, inter-individual immune responses that could logically be expected from field trapped animals that are not clonal and, therefore, have different antigen presentation capabilities due to their MHC (major histocompatibility complex) polymorphism. A common feature between males and females was the presence in the pre-immune sera of antibodies recognizing sperm proteins, as visible in Figure 1 and Figure 2. These proteins were probably not specific to the sperm lineage and they triggered immune responses due to inter-individual polymorphisms. The intensity of this response was weak, as confirmed by the very faint staining observed when performing an immunofluorescence on spermatozoa with the pre-immune sera, as shown in supplementary Figure S3.

Of the 120 *ATS* sperm antigens identified in this study, 15 were found to be strictly limited to the sperm lineage, most of which were described as plasma membrane-integrated or extracellular components. Using the power of the four replicates performed in the LC-MS/MS analysis, the number of sperm antigens capable of eliciting significant immune responses decreased to 22. The analysis of these 22 proteins using the “STRING” database, and considering the “cell compartments”, showed the three major categories were sperm parts (Table 4), thus strengthening the fact that an efficient and sperm-specific immune response was triggered by the subcutaneous injection of whole spermatozoa. When considering a threshold value of 2 for the mean fold change after immunization, a number of 91 proteins elicited a response. This means that some proteins, even though not showing a statistical significance due to the experimental design, may be efficient targets. The case of the protein CHDH is an illustration of that as the mFC was 21.01 for the males and 20.78 for the females, but it was statistically significant only for the males (Table S2). This study revealed that a selected peptide of the CHDH protein is indeed immunogenic when injected subcutaneously in male *ATS*. Further investigations on other proteins will be needed to determine their potential as a contraceptive target. This work is currently in progress. 

Twenty-nine proteins have already been identified in the epididymosomes of cattle [49,50], mice [51], or men [52], as reported in Table 2. Epididymosomes are small apocrine secretory vesicles emanating from the epididymal epithelium [53]. They function as cargo ships between the epididymal epithelium and maturing spermatozoa, transporting mainly proteins, lipids, and small RNAs. Immunizing *ATS* against some of these proteins (listed in Table 2) could be of interest because it could have two consequences. The first would be to target sperm antigens added to the sperm cells during the post-testicular phases (thus avoiding testis-specific antigens) and the second would be to potentially impede the transfer from the epididymosomes to the spermatozoa. We believe that this is very possible because the epithelium of the caput epididymis is suspected to be highly permeable to the systemic compartment, offering the opportunity to antibodies to reach the epididymal lumen and, consequently, the epididymosomes and spermatozoa [54,55].

Among the identified sperm proteins, YWHAZ or protein 14-3-3 ζ, also known as tyrosine 3-monooxygenase/tryptophan 5-monooxygenase activation protein zeta, a member of the YWHA family of adaptor proteins, particularly caught our attention. It is present in human [52] and mouse epididymosomes (M. Whitfield, personal communication) and it is a major protein for sperm functions, as described in bovine studies [56]. We previously showed that disorders of the mouse sperm capacitation process could be associated with decreased sperm YWHAZ content in a pathological situation such as dyslipidemia [57]. As YWHAZ function is central for male fertility, if the *ATS* YWHAZ protein contains an *ATS*-specific epitope with immunogenic potential, it could be considered as a contraceptive antigen worth testing. 

The production levels of ASA against some of the identified sperm antigens have already been reported in different species and under different circumstances. In male deer, the production of mucosal sperm-reactive antibodies against ACR, PGK2, and ENO1 was described [58]. Antibodies against ENO1, ALDOA, GAPDH, and ODF1 were also detected in human semen from infertile men [59]. Serum antibodies against GAPDHS were reported in higher concentrations in infertile men and women as compared to fertile individuals [60]. Three of these proteins (ACR, GAPDHS, and ODF1) are strictly present on spermatozoa, making them good sperm-cell-specific antigens. Among the sperm antigens identified, several are known to be involved in key steps of the fertilization process, such as HSPA1L [61], CCT3 [62], ZPBP and ZPBP2 [63], and HSPD1 [64,65]. Others, such as AKAP3 and ROPN1, are associated with the acrosomal reaction process [66,67]. These sperm antigens, because of their involvement in major fertilization events, also represent good vaccine targets. 

Male *ATS* immunized against two species-specific peptides of the CHDH (choline dehydrogenase) and the ZPBP2 (zona pellucida-binding protein 2) proteins developed specific immune responses, confirming that these two proteins are interesting targets. The immune sera obtained after CHDH immunization contained IgG that could bind both the midpiece and the acrosome region of *ATS* spermatozoa.

In conclusion, the immunization of male and female *ATS* with whole spermatozoa has allowed the identification of a significant number of potential sperm immunogenic antigens. Sex specificity of immune responses was observed both quantitatively and qualitatively. This paves the way for the development of a multi-antigen vaccination strategy to optimally target male and female partners. Some of the sperm antigens identified here are already known to provoke the generation of anti-sperm antibodies that interfere with reproductive success in various species. Others have been identified here for the first time, further expanding the options for developing a multi-targeted vaccine. The next step towards the generation of this vaccine in *ATS* will be to select, on these sperm antigens, immunogenic peptidic epitopes with sufficient sequence specificity to limit their immunogenicity to *ATS* so as to limit possible collateral effects on off-target fauna. Ultimately, one of the challenges will be to find the most effective route of administration of such a vaccine that could provide an alternative to the current chemical strategy used to control *ATS* outbreaks. Finally, the strategy used in this work represents a proof of concept that can be transposed on other wild harmful or invasive species to identify sperm antigens to be used for contraceptive vaccines with the aim of controlling their proliferation.

## 4. Materials and Methods

### 4.1. Animals 

Male and female *Arvicola terrestris Scherman* (*ATS*) were collected in open fields in the region of Puy-de-Dôme (France): Perpezat (45°68’33’’ N–2°78′33′’ E); Nébouzat (45°42’59’’ N–2°54′19′’ E); Saint-Julien-Puy-Lavèze (45°39’58’’ N–2°40′25′’ E). In the immediate vicinity of freshly made molehills, galleries were detected with a sounder and a trap was placed inside. Only animals of reproductive age, that is with a weight greater than 70 g, were kept alive and brought back to the animal facility where they were housed in a controlled environment (23 °C, 12 h light/12 h dark). The *ATS* were fed ad libitum with carrots. All the following procedures were approved by the Auvergne Animal Experiment Ethics Committee (C2E2A) and the French Ministry for Research (APAFIS authorization # 10653-2017071016422159 v5). The animals were anesthetized using 4% isoflurane (Isovet) in the inhaled air to perform the injections and blood sampling. They were then killed by cervical dislocation before tissue sampling. 

### 4.2. Production of Horseradish Peroxidase (HRP)—Conjugated Rabbit Anti-ATS Immunoglobulin G (IgG) Antibodies

To perform Western blot and immunofluorescence analyses, the production of a specific antibody directed against immunoglobulin G (IgG) from *ATS* was crucial. As mentioned above, blood was retrieved from anesthetized adult males and females *ATS* by intracardiac puncture and incubated overnight at 4 °C. Blood was centrifuged (1700× *g*, 20 min, 4 °C) to collect the IgG-containing serum. Briefly, *ATS* IgG samples were purified using protein A/G columns. Two rabbits were immunized with these purified IgG. Immunizations were carried out at day 0 (primo-injection) and at day 7 (booster #1), day 14 (booster #2), and day 34 (booster #3). Sera were then collected on day 63 by exsanguination, and rabbit IgG were purified using A-protein columns before being conjugated to horseradish peroxidase (HRP). The biotech company Agrobio (La Ferté-Saint-Aubin, France) produced this secondary antibody.

### 4.3. Animal Immunization Procedure 

Whole *ATS* spermatozoa (15 × 10^7^ spz/injection in a total volume of 300 µL, corresponding to an immunogenic dose based on protein content) were injected subcutaneously between the two shoulder blades of male and female *ATS*. Before spz injection, a blood fraction was collected in the tail vein to retrieve the pre-immune sera. Five males and six females were simultaneously immunized with whole spz. The immunization procedure lasted 6 weeks: on the first day, spz (15 × 10^7^) were injected with an equal volume of complete Freund’s adjuvant (Sigma, Saint-Quentin Fallavier, France) corresponding to primo-injection. After three weeks, the same spz quantity was injected, diluted in an equal volume of incomplete Freund’s adjuvant (booster #1). The immunization procedure was stopped after six weeks and blood was collected by intracardiac puncture under anesthesia, as described above, to retrieve the immune sera. Control immunizations were performed by injecting Freund’s adjuvant alone diluted in PBS. Animals were sacrificed by cervical dislocation. The time of vaccination was determined based on a proof-of-concept study [34]. 

### 4.4. Sperm Collection

For all experiments, spz were collected by pressure on cauda epididymis in 500 µL of Whitten’s HEPES buffer (WH: 100 mM NaCl, 4.7 mM KCl, 1.2 mM KH_2_PO_4_, 1.2 mM MgSO_4_, 5.5 mM glucose, 1 mM pyruvic acid, 4.8 mM lactic acid, 20 mM HEPES, pH 7.4). After 30 min at 37 °C to release a maximum of spz from the cauda epididymis, spz were retrieved, an aliquot was diluted (1:50 in WH), and they were counted using a Malassez hemacytometer. They were finally washed three times by centrifugation in PBS (500× *g*, 5 min, room temperature—RT) and the final pellet was frozen and kept at −20 °C until use.

### 4.5. Extraction of Sperm Proteins

For mono-dimensional SDS-PAGE analyses, the spz from cauda epididymides were incubated in RIPA buffer for 2 h at RT (50 mM Tris, 150 mM NaCl, 12 mM sodium deoxycholate, 3 mM sodium dodecyl sulfate, 1% Igepal CA-630) supplemented with protease inhibitors (Complete Mini, Roche Diagnostics, Meylan, France) and phosphatase inhibitors (Halt Phosphatase Inhibitor Cocktail, Thermo Scientific, Waltham, MA, USA). Samples were then sonicated for 30 min with a 30 s/30 s cycle at 4 °C (Bioruptor UCD-200TM-EX, Diagenode, Liege, Belgium). Cell lysates were centrifuged (12,000× *g*, 10 min, 4 °C) and the supernatants containing easily solubilized proteins were recovered for the determination of protein concentrations by the Bradford assay (Biorad, Hercules, CA, USA) with bovine serum albumin (BSA, Euromedex, Souffelweyersheim, France) as the standard. Samples were then frozen at −20 °C until analyses. 

For two-dimensional electrophoresis analyses, spz proteins were extracted using the commercially available ReadyPrep protein extraction kit (BioRad). This kit allows the preparation of total cellular protein extracts using strong chaotropic agents such as the zwitterionic detergent ASB-14. Briefly, spz were resuspended (2 h, RT) in lysis buffer (7 M urea, 2 M thiourea, 1% (*w/v*) ASB-14 detergent, 40 mM Tris base, 0.001% Bromophenol Blue, 200 mM tributylphosphine, Bio-Lyte 3/10) and supplemented with protease inhibitors and phosphatase inhibitors. Samples were sonicated for 30 min (30 s/30 s) at 4 °C and then centrifuged at RT (16,000× *g*, 20 min) to remove insoluble material. The protein concentration was determined using the bicinchoninic acid assay (Biorad) with BSA as standard.

### 4.6. Serum Globulin Profile with Amido Black Staining

Agarose gel electrophoresis was carried out (180 V, 2 h) on different sera from *ATS* to compare the immune and pre-immune sera of each animal. Total serum proteins were mixed with alkaline veronal buffer, pH 8.2 (5,5-Diethylbarbituric acid sodium salt) at the dilution ¼ and supplemented with Green GoTaq Flexi Buffer (Promega, Madison, WI, USA). Total serum proteins were separated on 1% agarose gels (Euromedex) in veronal buffer. The different fractions of *ATS* serum proteins were visualized by direct coloration with amido black 10B (4-Amino-5-hydroxy-3-[(4-nitrophenyl)azo]-6-(phenylazo)-2,7-naphthalene disulfonic acid, Merck, Lyon, France). The gels were fixed for 2 h with a methanol/glacial acetic acid/distilled water solution (volume ratio 4/1/4), then stained for 15 min with the staining solution (amido black: 1.25 g dissolved in 5% acetic acid), and, finally, discolored for 2 days in a bleaching solution (acetic acid 5%).

### 4.7. Western Blot Analysis

Thirty µg of spz proteins were loaded onto 12% SDS-PAGE gel. After migration, proteins were transferred on PVDF membranes (Hybond ECL, Amersham Biosciences, Sigma) activated in absolute ethanol (10 min, RT). Membranes were blocked (1 h, RT) with Tris-buffered-saline (TBS) (50 mM Tris, 150 mM NaCl) containing 0.1% *v/v* Tween-20 (TBS-T) and 5% *w/v* BSA. Membranes were hybridized in TBS-T, 2% *w/v* BSA with either *ATS* immune or pre-immune serum (dilution 1:1000, overnight, 4 °C) or with rabbit anti-GAPDH (1:10,000, Sigma). Membranes were then washed three times with TBS-T (10 min, RT) and further hybridized with HRP-conjugated rabbit anti-*ATS* IgG antibody (Agrobio) or HRP-conjugated anti-rabbit antibody for GAPDH (BI 2407, dilution 1:5000, Abliance, Compiègne, France) for 1 h at RT. Membranes were finally washed thrice with TBS-T (3 × 10 min). The protein bands were detected with chemiluminescent reagents (Clarity Western ECL Substrate, Biorad) using a ChemiDoc MP Imaging System (Biorad).

### 4.8. Two-Dimensional Electrophoresis Analysis

IsoElectric focusing (IEF) was performed on IPG 11 cm long (pH 3–10) strips (Biorad) using 200 μg of proteins. IEF was carried out using a PROTEAN IEF Cell (Biorad) as follows: passive rehydration (10 h), active rehydration at 50 V (12 h), and migration (250 V for 15 min, 8000 V for 2h30, and a final phase of 8000 V up to a maximum of 35,000 V/h). Following IEF, the focused strips were rotated in 135 mM dithiothreitol (Euromedex) in equilibration buffer (6 M urea; 2% SDS; 0.375 M Tris–HCl pH 8.8; 20% glycerol, 10 mL for 15 min) and, subsequently, in 135 mM iodoacetamide (Sigma) in equilibration buffer (10 mL for 15 min). Second-dimension gels (1 mm thick, 4–15% gradient acrylamide, Criterion TGX—IPG 11cm+1well precast gels, Biorad) were run (5 h, 80 V) using the criterion cell (Biorad). Proteins were then transferred on activated PVDF membranes and blocked with TBS-T, 5% *w/v* BSA. Membranes were hybridized with pre-immune or immune serum (1:1000, overnight, 4 °C), anti-beta-actin (1:2500, Sigma), or anti-GAPDH (1:10,000, Sigma) and secondary antibodies were applied after three washing steps in TBS-T: rabbit HRP-conjugated anti-*ATS* IgG antibody (1:1000, 1 h, RT) or HRP-conjugated anti-rabbit IgG antibody (BI 2407, 1:1000 for beta-actin and 1:5000 for GAPDH, Abliance). Protein spots were finally revealed by ECL as described above. 

### 4.9. Direct Immunofluorescent Labelling of Sperm Cells

Before experiments, slides (Superfrost, Thermo Scientific) were treated with acetone (45 min, RT) and stored in absolute ethanol (−20 °C) until use. The cauda epididymis of non-immunized *ATS* were perforated with a needle in WH buffer and incubated for 30 min at RT. Spz were recovered by centrifugation (500× *g*, 5 min), washed thrice with PBS, smeared onto slides, and allowed to air dry (1 h, RT). Slides were then washed twice for 5 min in PBS without shaking. Saturation was performed with PBS containing 0.1% *w/v* BSA (1 h, RT) and the slides were incubated with pre-immune or immune serum (1:1000, overnight, 4 °C) in a solution of PBS, 0.01% Tween-20 (PBS-T) containing 0.1% *w/v* BSA. As fluorescent secondary antibodies directed against *ATS* IgG are not available, slides were incubated with a rabbit anti-IgG *ATS* antibody (1:1000, Agrobio) in PBS, 0.1% BSA (1 h, RT) before applying an Alexa Fluor 555-conjugated goat anti-rabbit IgG antibody (1:1000, 1 h, RT, Invitrogen, Thermo Scientific). After two washes, slides were incubated in PBS-T, 0.1% *w/v* BSA either with lectin-PNA from Alexa Fluor 488-conjugated *Arachis hypogaea* agglutinin (1:20, 30 min, 37 °C, Invitrogen) or with MitoTracker Green FM Dye (1:5000, 30 min, 37 °C, Invitrogen). After two washes with PBS, nuclei were stained with Hoechst 33342 (1 µg/mL in PBS, 5 min in the dark, Invitrogen). Slides were finally mounted in a Citifluor AF100 anti-fading solution and Citifluor Tris-MWL 4–88 (ratio 1:9 respectively, Electron Microscopy Sciences, Hatfield, PA, USA) and stored at +4 °C until observation by confocal microscopy.

### 4.10. Confocal Microscopy 

The acquisition of triple staining was done using a Leica SP8 laser scanning confocal microscope, equipped with a Plan Apo λ 40X Oil objective, with a pinhole size of 1 AU (arbitrary unit), Z-step: 0.15 µm. The parameters used for the channels series with lectin-PNA Alexa Fluor 488 conjugate were as follows: Channel 1 (Laser 405 nm): Hoechst 33342 detection window between 430 nm and 531 nm using a Hybrid detector at a gain of 10%. Channel 2 (laser 488 nm): lectin-PNA Alexa Fluor 488: detection window 493 nm–541 nm, using a Hybrid detector at a gain of 10% gain. Channel 3 (laser 552 nm): Alexa Fluor 555 detection window 569 nm–621 nm using a PMT detector at a gain of 700 V.

The parameters used for the channels series with MitoTracker Green FM Dye were as follows: Channel 1 (Laser 405 nm): Hoechst 33342 detection window between 430 nm and 531 nm using a Hybrid detector at a gain of 15%. Channel 2 (laser 488 nm): PNA Alexa Fluor 488: detection window 493 nm and 541 nm, using a Hybrid detector at a gain of 10%. Channel 3 (laser 552 nm): Alexa Fluor 555: detection window between 569 nm and 621 nm using a PMT detector at a gain of 700 V. Z-stack images were taken through representative spz to enable projected image reconstruction using an image analysis software Imaris 7.6 (Bitplane AG, Scientific Software, Zurich, Switzerland). Images were reconstructed in 3D form using isosurface rendering to determine the nature of the relationship between immunolabeling markers. These images’ acquisitions were carried out at the Confocal Microscopy Facility CLIC (Clermont Imagerie Confocale) at Université Clermont Auvergne, iGReD Laboratory (France).

### 4.11. Indirect Immunoprecipitation Assays

Spz from cauda epididymis were collected as described earlier, and 10 × 10^7^ spz cells were incubated in 1.2 mL RIPA buffer with protease and phosphatase inhibitors (2 h, RT). After sonication as described earlier, lysates were centrifuged (12,000× *g*, 10 min, 4 °C). One hundred µL samples of supernatants were incubated with protein A/G magnetic beads (30 min, 4 °C, Dynabeads Protein G, Thermo Scientific) for pre-clearing. Then, this pre-clearing supernatant was incubated with 25 µL of either immune serum or pre-immune serum with slight rotation (7 rpm) overnight at 4 °C (to limit proteolysis). Supernatants (now containing antibodies–antigen complexes) were incubated with protein A/G magnetic beads (4.5 mg/mL, Thermo Scientific) with slight rotation (2 h, RT). The beads–antibodies–antigen complexes were eluted with 35 µL of elution buffer (50 mM glycine, pH 2.8) for 20 min at RT in order to detach the beads from the Ab-Ag complexes. On one hand, protein A/G magnetic beads (4.5 mg/mL) were again added to separate antibodies and antigens. The antigen-containing supernatants were mixed with Laemmli 4× buffer and boiled at 95 °C for 10 min. On the other hand, magnetic beads were washed thrice with 200 µL of PBS and boiled at 95 °C for 10 min in 25 µL of Laemmli 4 × buffer before SDS-PAGE Western blot as described above.

### 4.12. In-Gel Trypsin Digestion 

Proteins obtained by immunoprecipitation (above) were migrated in a stacking polyacrylamide gel (12%) and the 1D bands (protein gel) of interest excised from the G250 Blue (Thermo Scientific)-stained SDS-PAGE gel (approximately 5 mm length fragments) were reduced with 200 µL of 10 mM Dithiothreitol (Euromedex) in 50 mM ammonium bicarbonate buffer (Sigma) at 56 °C for 1 h. Then, it was followed by the alkylation with 55 mM iodoacetamide (Sigma) in the same buffer at RT for 30 min in the dark. Then, the protein gels were discolored with 100 µL of 5% (15 min) and 50% (30 min) acetonitrile successively in 25 mM ammonium bicarbonate buffer. They were then dehydrated by incubation in 200 μL of 100% acetonitrile for 10 min. The protein gel was digested by an overnight incubation with 100 µL of trypsin solution (10 ng/µL, Promega) in 25 mM ammonium bicarbonate buffer at 37 °C. Resulting peptide mixtures were extracted with 50% acetonitrile in 20 mM ammonium bicarbonate buffer. Finally, they were concentrated with a speed vacuum. The volumes were adjusted to 15 µL with 98% H_2_O solution/2% acetonitrile/0.05% trifluoroacetic acid, if the volume was less than 15 µL for further analysis by LC-MS/MS.

### 4.13. LC-MS/MS Analysis and Identification of Antigens

The proteins obtained from the immunoprecipitation assays and hydrolyzed by trypsin were submitted to LC-MS/MS analysis. Six microliters of each hydrolysate were injected into the nanoHPLC chain (Ultimate 3000, Dionex, Sunnyvale, CA, USA) on a concentration column to retain the peptides and remove contaminants that could be disruptive in the mass spectrometry analysis. The peptides were then separated according to their hydrophobicity on an analytical column (Acclaim 75 µm, 15 cm pepmap C18, 2micro 100 A PN16453, SN10702989). NanoHPLC was coupled to a nanoESI source, a mass-spectrometer-type Q-Orbitrap HFX (Thermo Scientific) that operates in data-dependent mode. At the end of the LC-MS/MS analysis, the samples were analyzed in the PROGENESIS QI software (v 4.0, Nonlinear Dynamics). This software allowed both the detection and quantification of all peptide ions detected in the analysis, then the MS/MS analyses performed in the mass spectrometer were interrogated in the *Microtus ochrogaster* database. As *ATS* does not have any available databank, *Microtus ochrogaster* was used due to its phylogenetic proximity. The query engine used was Mascot (V. 2.5.1, internal license version) and Peaks (v10, internal license version). Protein abundance was calculated from the sum of the abundances of the normalized area of each peptide for a specific protein for each run. As the identification was carried out by the Mascot and Peaks software, proteins with high-confidence (FDR < 0.01) were considered as positively identified proteins. These proteomic experiments and analyses were carried out at the INRAe facility (Plateforme d’Exploration du Métabolisme, Composante Protéomique—PFEMcp), Theix, France.

### 4.14. Slot Blot Analysis

The presence of serum IgG directed against the injected peptides was evaluated by slot blot. Increasing quantities of the peptides used for immunization were slotted on nitrocellulose membranes, which were then processed as described above for the Western blot procedure. Pre-immune and immune sera were used after dilution to 1/500 in TBS-T, 2% *w/v* BSA. 

### 4.15. Immunization of Male ATS with Selected Peptides

Two candidate proteins were chosen to test their ability to trigger a specific immune response in male *ATS*: CHDH and ZPBP2. The former was chosen because it gave high responses in both males and females, and the latter because it is sperm-specific and involved in gamete interaction (see Table S2). One peptide for each protein was selected based on two main criteria: the species specificity and the potential immunogenicity (depending on their hydrophobicity and on their amino-acid sequence). The chosen sequences remain confidential. Peptides were synthesized (Eurogentec, Seraing, Belgium) and, for each peptide, 500 µg were injected subcutaneously in 3 male *ATS*, as described for whole spermatozoa. A “boost” was made after 3 weeks and the immune sera were collected after 7 weeks (a sample of pre-immune serum was collected for each individual before the first injection). Pre-immune and immune sera were analyzed by slot blot and immunofluorescence. 

### 4.16. Statistical Analysis

The maximum fold change (mFC) of proteins detected with male or female immune sera was compared with the mFC of proteins detected for male or female pre-immune sera. The statistical analyses were performed with a Mann–Whitney test performed using the R software.

### 4.17. Bioinformatics

The protein–protein interaction network for the identified proteins was annotated using the STRING v11 database (recurrent instance search tool for neighboring genes at http://www://string-db.org/ (accessed on 7 July 2020)).

## Figures and Tables

**Table 1:** Proteins recognized by ASA, produced by male and female *ATS*, and identified by LC-MS/MS. The classification was made according to their function in the reproductive process or their subcellular location.

Protein Name	UniProtKB IDs	MW (Da)	Th.Ip	mFC Male	mFC Female	No. of Peptides (% Coverage)	Mascot Score
Sperm–egg interaction							
ALDOA	P05064	37,817	8.30	3.75	4.33	8 (43.20)	497.32
HSPA1L *	P16627	70,981	5.76	6.83	25.86	2 (7.80)	234.52
CCT3	P80318	61,265	6.10	5.86	89.02	2 (3.49)	79.20
CCT8	P42932	60,116	5.42	1.52	1.81	2 (4.38)	123.39
ZPBP *	Q62522	42,640	9.43	2.83	6.57	7 (15.26)	386.51
ZPBP2 *	Q6X786	42,640	8.04	2.83	6.57	7 (22.92)	580.50
HSPD1 (HSP60)	P63038	55,970	5.70	1.79	6.83	7 (19.01)	397.55
Penetration of zona pellucida							
HEXB	P20060	54,911	6.29	1.96	4.07	5 (12.82)	319.44
ACR *	P23578	50,286	9.26	1.65	3.34	6 (18.88)	468.93
Sperm capacitation							
PEBP1	P70296	21,024	7.01	3.10	5.77	3 (28.33)	198.37
AKAP3 *	O88987	94,787	5.84	3.52	12.72	3 (3.20)	169.17
YWHAZ	P63101	27,898	4.73	1.27	2.65	7 (30.20)	503.69
PRKACA	P05132	40,713	8.84	0.95	0.49	6 (14.29)	322.06
ROPN1 *	Q9ESG2	24,123	5.52	1.51	7.34	3 (18.90)	233.78
Acrosomal reaction							
CALR	P14211	48,159	4.29	1.76	3.41	11 (28.92)	864.52
PDIA3	P27773	57,052	5.98	1.94	5.29	12 (26.14)	770.66
ACR *	P23578	50,286	9.26	1.65	3.34	6 (18.88)	468.93
HEXB	P07686	54,911	6.29	1.96	4.07	5 (12.82)	319.44
AKAP3 *	O88987	94,787	5.84	3.52	12.72	3 (3.20)	169.17
PRKACA	P05132	40,713	8.84	0.95	0.49	6 (14.29)	322.06
Sperm part							
ALDOA	P05064	37,817	8.30	3.75	4.33	8 (43.20)	497.32
NUP210L *	Q9D2F7	202,450	7.15	5.64	6.87	33 (17.50)	2493.12
PRKAR2A	P12367	45,817	4.96	1.52	3.41	3 (7.37)	167.54
ODF1 *	Q61999	29,282	8.46	1.57	2.01	23 (53.31)	1421.42
GAPDHS *	Q64467	35,936	8.39	1.51	2.86	8 (9.36)	178.10
PGK2 *	P09041	27,881	8.74	1.33	2.77	4 (6.95)	233.10
OAZ3*	Q9R109	26,958	6.38	8.10	20.86	2 (7.69)	202.45
AKAP3 *	O88987	94,787	5.84	3.52	12.72	3 (3.20)	169.17
DYNLL2	Q9D0M5	10,457	6.81	2.79	4.45	2 (27)	199.68
ROPN1 *	Q9ESG2	24,123	5.52	1.51	7.34	3 (18.90)	233.78
PRKACA	P05132	40,713	8.84	1.06	2.05	6 (14.29)	322.06
Carbohydrate metabolism/Glycolysis and Gluconeogenesis							
ALDOA	P05064	37,817	8.30	3.75	4.33	8 (43.20)	497.32
ACO2	Q99KI0	86,107	7.36	1.68	3.84	14 (21.50)	1013.27
GAPDH	P16858	35,936	8.57	1.51	2.86	8 (31.23)	594.17
PGK1	P09411	27,881	8.30	1.33	2.77	4 (11.37)	381.29
PGK2 *	P09041	27,881	8.74	1.33	2.77	4 (6.95)	233.10
MDH2	P08249	36,045	8.92	2.78	7.06	18 (64)	1260.95
CS	Q9CZU6	52,010	8.45	3.57	7.15	8 (21.72)	699.94
ENO1	P17182	47,602	7.01	1.86	2.40	9 (23.04)	511.79
GPI1 (G6PI)	P06745	62,986	8.42	2.07	3.05	4 (9.32)	245.83
SUCLA2	Q9Z2I9	50,481	7.05	1.32	2.22	7 (33.81)	803.25
MTHFD1L	Q3V3R1	105,743	8.32	3.81	6.45	2 (3.10)	132.20
LDHC *	P00342	28,302	7.08	1.30	0.83	3 (17.76)	323.87
HK3	Q3TRM8	102,184	5.23	0.39	7.61	3 (3.15)	163.23
Energy metabolism							
PRDX5	P99029	20,423	6.00	1.91	4.29	5 (17.60)	313.59
CAR1	P13634	28,550	6.59	1.72	3.44	9 (52.17)	686.90
CAR2	P00920	29,294	6.87	1.11	2.21	4 (17.31)	233.50
SDHA	Q8K2B3	73,414	7.06	1.49	5.47	7 (11)	393.12
ATP5B	P56480	25,127	5.26	1.27	2.67	15 (25.39)	1084.34
ATP5F1	Q9CQQ7	10,909	9.37	0.97	1.87	2 (7.11)	111.97
COX2	P00405	26,025	4.67	0.96	2.01	5 (16.30)	311.67
COX4I1	P19783	10,899	9.52	0.78	1.90	6 (41.30)	357.12
ATP5A1	Q03265	58,502	9.16	2.47	5.22	14 (25.28)	1072.18
Mitochondrial protein							
PRDX3	P20108	28,428	7.68	2.07	4.46	2 (7.66)	115.88
MTIF2	Q91YJ5	10,909	6.71	2.82	7.30	7 (26.91)	488.06
COX2	P00405	26,025	4.67	0.96	2.01	5 (16.30)	311.67
SUCLA2	Q9Z2I9	50,481	7.05	1.32	2.22	7 (33.81)	803.25
DIABLO	Q9JIQ3	26,705	5.68	2.95	404.15	2 (8.09)	83.35
CHDH	Q8BJ64	67020	8.57	21.01	20.77	3 (4.86)	164.31
SDHA	Q8K2B3	73,414	7.06	1.49	5.47	7 (11)	393.12
ATP5B	P56480	25,127	5.26	1.27	2.67	15 (25.39)	1084.34
ATP5F1	Q9CQQ7	10,909	9.37	0.97	1.87	2 (7.11)	111.97
COX4I1	P19783	10,899	9.52	0.78	1.90	6 (41.30)	357.12
COX6B2	Q80ZN9	10,835	9.21	0.85	1.58	2 (23.86)	100.88
NIPSNAP3B	Q9CQE1	28,368	9.21	0.77	1.77	3 (22.84)	213.10
HSPA1L *	P16627	70,981	5.76	6.83	25.86	2 (7.80)	234.52
HSPD1 (HSP60)	P63038	55,970	5.70	1.79	6.83	7 (19.01)	397.55
ATPIF1	O35143	12,255	4.82	2.91	19.66	2 (17.76)	105.38
CYCT *	P00015	11,654	9.54	2.56	10.67	2 (27.62)	776.30
CYCS	P62897	11,654	9.54	2.56	10.67	2 (8.92)	250.71
ATP5A1	Q03265	58,502	9.16	2.47	5.22	14 (25.28)	1072.18
MTHFD1L	Q3V3R1	105,743	8.32	3.81	6.45	2 (3.10)	132.20
Chaperones							
HSPA5 (GR78/BIP)	P20029	72,606	5.07	1.99	4.19	21 (31.76)	1701.29
HSC70 (HSPA8)	P63017	71,069	5.37	3.99	4.49	5 (16.10)	520.24
HSPA1L *	P16627	70,981	5.76	6.83	25.86	2 (7.80)	234.52
HSPD1 (HSP60)	P63038	55,970	5.70	1.79	6.83	7 (19.01)	397.55
HSP90B1	P08113	92,865	4.76	1.19	1.95	16 (31.49)	1123.06

The UniProtKb ID, molecular weight (MW), and theoretical isoelectrical point (Th.Ip) are indicated for each protein. The number of peptides and the percentage of coverage, as well as the mascot score are also indicated. For the mascot score, only the peptides for which the score was greater than or equal to that of a validation with less than 1% false positive (FDR 1%) were retained. The mean fold change (mFC) obtained from male and female samples is reported. The stars (*) indicate proteins strictly expressed in spermatozoa.

**Table 2:** Proteins recognized by ASA belonging to three special categories: moonlighting proteins, proteins giving a phenotype limited only to the sexual organs when the expression of their gene is invalidated, and proteins contained in the epididymosomes.

Protein Name	UniProtKB IDs	MW (Da)	Th.Ip	mFc Male	mFc Female	No. of Peptides (% Coverage)	Mascot Score
Moonlighting proteins							
PGK1	P09411	27,881	8.30	1.33	2.77	4 (11.37)	381.29
PGK2 *	P09041	27,881	8.74	1.33	2.77	4 (6.95)	233.10
GAPDH	P16858	35,936	8.57	1.51	2.86	8 (31.23)	594.17
ACO2	Q99KI0	86,107	7.36	1.68	3.84	14 (21.5)	1013.27
ALDOA	P05064	37,817	8.30	3.75	4.33	8 (43.20)	497.32
ODF1 *	Q61999	29,282	8.46	1.57	2.01	23 (53.31)	1421.42
HSPA5	P20029	72,606	5.07	1.99	4.19	21 (31.76)	1701.29
ACR *	P23578	50,286	9.26	1.65	3.34	6 (18.88)	468.93
ENO1	P17182	47,602	7.01	1.86	2.40	9 (23.04)	511.79
HSPD1 (HSP60)	P63038	55,970	5.70	1.79	6.83	7 (19.01)	397.55
Phenotype limited only to sexual organs when the gene expression is invalidated							
NUP210L *	Q9D2F7	202,450	7.15	5.64	6.87	33 (17.50)	2493.12
PGK1	P09411	27,881	8.30	1.33	2.77	4 (11.37)	381.29
PGK2 *	P09041	27,881	8.74	1.33	2.77	4 (6.95)	233.10
ODF1 *	Q61999	29,282	8.46	1.57	2.01	23 (53.31)	1421.42
GAPDHS *	Q64467	35,936	8.39	1.51	2.86	8 (9.36)	178.10
ACR *	P23578	50,286	9.26	1.65	3.34	6 (18.88)	468.93
AKAP3 *	O88987	94,787	5.84	3.52	12.72	3 (3.20)	169.17
OAZ3 *	Q9R109	26,958	6.38	8.10	20.86	2 (7.69)	202.45
HSPA1L *	P16627	70,981	5.76	6.83	25.86	2 (7.80)	234.52
ZPBP *	Q62522	42,640	9.43	2.83	6.57	7 (15.26)	386.51
ZPBP2 *	Q6X786	42,640	8.04	2.83	6.57	7 (22.92)	580.50
CHDH	Q8BJ64	67,020	8.57	21.01	20.77	3 (4.86)	164.31
ROPN1 *	Q9ESG2	24,123	5.52	1.51	7.34	3 (18.90)	233.78
PRKACA	P05132	40,713	8.84	0.95	0.49	6 (14.29)	322.06
Epididymosomes							
HSC70 (HSPA8)	P63017	71,069	5.37	3.99	4.49	5 (16.10)	520.24
CAR1	P13634	28,550	6.59	1.72	3.44	9 (52.17)	686.90
PEBP1	P70296	21,024	7.01	3.10	5.77	3 (28.33)	198.37
TUBB4B	P68372	50,255	4.79	2.27	2.14	15 (63.82)	2370.87
ALDOA	P05064	37,817	8.30	3.75	4.33	8 (43.20)	497.32
CALR	P14211	48,159	4.29	1.76	3.41	11 (28.92)	864.52
HSPA5	P20029	72,606	5.07	1.99	4.19	21 (31.76)	1701.29
PRDX5	P99029	20,423	6.00	1.91	4.29	5 (17.60)	313.59
HEXB	P20060	54,911	6.29	1.96	4.07	5 (12.82)	319.44
PGK1	P09411	27,881	8.30	1.33	2.77	4 (11.37)	381.29
PRKAR2A	P12367	45,817	4.96	1.52	3.41	3 (7.37)	167.54
PDIA3	P27773	57,052	5.98	1.94	5.29	12 (26.14)	770.66
GAPDH	P16858	35,936	8.57	1.51	2.86	8 (31.23)	594.17
GAPDHS *	Q64467	35,936	8.39	1.51	2.86	8 (9.36)	178.10
PRDX1	P35700	22,309	8.27	2.21	4.55	4 (31.30)	344.45
PRDX2	Q61171	22,163	5.66	1.50	2.93	3 (15)	230.67
PRDX6	O08709	23,741	6.00	2.75	5.31	2 (8.93)	96.41
AKR1B3	P45376	36,056	6.52	2.09	3.59	2 (9.05)	97.03
AKAP3 *	O88987	94,787	5.84	3.52	12.72	3 (3.20)	169.17
YWHAZ	P63101	27,898	4.73	1.27	2.65	7 (30.20)	503.69
ENO1	P17182	47,602	7.01	1.86	2.40	9 (23.04)	511.79
CCT3	P80318	61,265	6.10	5.86	89.02	2 (3.49)	79.20
HSPD1 (HSP60)	P63038	55,970	5.70	1.79	6.83	7 (19.01)	397.55
ZPBP *	Q62522	42,640	9.43	2.83	6.57	7 (15.26)	386.51
ZPBP2 *	Q6X786	42,640	8.04	2.83	6.57	7 (22.92)	580.50
HSP90B1	P08113	92,865	4.76	1.20	2.09	16 (31.49)	1123.06
PPIA	P17742	18,089	7.68	3.05	7.45	2 (17.83)	206.22
PDIA4	P08003	72,914	4.96	4.75	4.94	4 (7.01)	217.37
RAB2A	P53994	23,545	6.08	0.27	0.32	3 (15.10)	152.70

The UniProtKb ID, molecular weight (MW), and theoretical isoelectrical point (Th.Ip) are indicated for each protein. The number of peptides and the percentage of coverage, as well as the mascot score, are also indicated. For the mascot score, only the peptides for which the score was greater than or equal to that of a validation with less than 1% false positive (FDR 1%) were retained. The mean fold change (mFC) obtained from male and female samples is reported. The stars (*) indicate proteins strictly expressed in spermatozoa.

**Table 3:** Proteins recognized by ASA belonging to additional categories.

Protein Name	UniProtKB IDs	MW (Da)	Th.Ip	mFc Male	mFc Female	No. of Peptides (% Coverage)	Mascot Score
DNA/RNA binding							
NUP155	Q99P88	156,682	5.78	7.96	3.18	5 (3.80)	297.33
PPIB	P24369	23,754	9.42	1.84	5.53	3 (15.74)	212.97
SERPINA3	Q91WP6	46,647	5.37	1.64	2.34	3 (11.87)	103.57
histone H3.1	P68433	15,509	10.31	2.93	3.98	5 (22.06)	285.16
EEF1A1	P10126	50,440	9.10	0.79	1.30	5 (10.82)	311
Histone H4	P62806	11,360	11.36	0.89	7.30	5 (55.34)	396.84
HIST1H1E	P43274	21,882	11.03	3.75	1.15	5 (23.30)	484.74
TXN1	P10639	11,952	9.59	1.26	2.07	2 (20.95)	122.51
PPIA	P17742	18,089	7.68	3.05	7.45	2 (17.83)	206.22
Hist1h2bc	Q6ZWY9	13,898	9.68	1.67	2.60	5 (28.57)	319.82
GRHL3	Q5FWH3	67,554	6.40	1.30	0.97	2 (2.66)	88.29
Complement activation							
C3	P01027	173,622	6.02	1.37	2.56	26 (48.50)	7024.70
C1QC	Q02105	27,193	8.61	1.39	0.85	22 (29.10)	1752.80
C1QB	P14106	27,193	8.83	1.73	0.99	54 (40.60)	4681.20
C4BP	P08607	53,951	6.83	0.85	1.05	7 (14.50)	614.73
CPN2	Q9DBB9	61,608	5.63	0.95	3.61	3 (4.94)	191.85
C2	P21180	144,278	7.23	32.07	4.11	20 (13.10)	1593.08
C1QA	P98086	26,093	9.26	1.35	0.87	17 (29.80)	1017.74
CFI	Q61129	69,853	7.72	1.47	2.11	8 (12.67)	551.94
Cytoskeletal protein							
ACTB	P60710	41,890	5.29	1.30	2.41	6 (14.29)	322.06
TUBA1C	P68373	50,566	4.96	1.34	2.49	2 (36.53)	1498.38
TUBB4B	P68372	50,255	4.79	2.27	2.14	15 (63.82)	2370.87
DSP	E9Q557	334,465	6.44	2.25	0.08	4 (1.25)	169.10
GFAP	P03995	49,987	5.42	0.70	0.26	1 (2.56)	33.58
JUP	Q02257	82,479	5.75	1.34	0.31	3 (4.56)	117.12
MSN	P26041	67,816	6.08	0.59	0.62	2 (2.77)	106.67
Lipid metabolism							
CES1	Q8VCC2	62,577	6.15	3.75	6.59	3 (8.85)	278.86
CRAT	P47934	71,289	8.63	3.52	8.07	10 (16.45)	605.83
PTGDS	O09114	21,523	7.66	2.23	6.21	2 (8.90)	124.98
NPC2	Q9Z0J0	17,000	7.57	1.77	4.37	4 (14.67)	198.99
EPHX1	Q9D379	52,808	6.77	4.86	Infinity	2 (3.74)	60.14
GK	Q8C635	58,210	6.12	0.83	2.96	2 (4.39)	135.99
GK2 *	Q9WU65	58,210	5.57	0.83	2.96	2 (4.11)	127.32
GK3 * (in human)	Q14409	58,210	6.00	0.83	2.96	2 (4.17)	129.18
APOE	P08226	35,619	5.65	1.08	2.31	19 (44.20)	1278.93
APOA4	P06728	44,043	5.28	1.03	2.23	31 (48.90)	2557.95
APOM	Q9Z1R3	21,771	5.66	4.92	1.79	2 (8.95)	109.44
APOC1	P34928	9893	8.01	1.07	6.05	2 (20.45)	104.77
PON1	P52430	39,785	5.08	0.46	0.58	6 (23.94)	458.68
ADIPOQ	Q60994	26,435	5.42	0.48	1.01	2 (11.11)	149.13
ECI1	P42125	32,546	8.80	2.68	1.44	2 (10.38)	149.01
Others							
RPS27A	P62983	12,358	9.34	1.73	3.20	9 (40.19)	503.74
ITIH1	Q61702	101,362	6.31	1.25	3.41	11 (17.5)	1101.71
AMBP	Q07456	39,481	5.95	1.85	4.09	2 (7.22)	153.34
TTR	P07309	15,833	11.13	1.27	2.33	15 (59.90)	1558.55
ERP44	Q9D1Q6	47,274	5.09	3.75	4.41	2 (4.93)	61.93
CPN2	Q9DBB9	49,092	7.61	1.05	3.61	2 (6.87)	105.60
PROC	P33587	52,746	5.85	1.84	3.37	2 (4.16)	84.11
TRF	Q921I1	78,371	6.81	1.09	2.13	94 (52.9)	9020.85
KNG1	O08677	38,818	6.34	0.84	1.53	3 (21.10)	872.77
ATRN	Q9WU60	162,969	7.24	0.76	2.91	2 (1.20)	80.25
ITIH3	Q61704	78,169	5.49	0.62	1.07	2 (13.50)	896.4
NMES1	Q810Q5	9589	9.45	0.72	1.12	2 (11)	71.69
CTSD	P18242	44,768	6.10	1.63	0.75	7 (14.2)	324.5
SERPINA1	P07758	58,642	5.37	7.14	6.26	3 (30)	103.57
GSTA3	P30115	22,658	9.21	9.08	1.81	2 (13.30)	148.63
Il4i1	O09046	72,032	8.79	2.33	4.16	5 (9.30)	236.71
SERPINA3F	Q80X76	45,927	4.91	1.37	1.83	8 (13.93)	882.80
PZP	Q61838	165,815	5.97	0.86	1.17	50 (21.78)	3570.34
PDIA4	P08003	72,914	4.96	4.75	4.94	4 (7.01)	217.37
RAB2A	P53994	23,545	6.08	0.27	0.32	3 (15.10)	152.70
CPB2	Q9JHH6	49,092	6.72	0.34	0.35	2 (3.55)	83.62

The UniProtKb ID, molecular weight (MW), and theoretical isoelectrical point (Th.Ip) are indicated for each protein. The number of peptides and the percentage of coverage, as well as the mascot score, are also indicated. For the mascot score, only the peptides for which the score was greater than or equal to that of a validation with less than 1% false positive (FDR <1%) were retained. The max fold change (mFC) obtained from male and female samples is reported. The stars (*) indicate proteins strictly expressed in spermatozoa.

**Table 4:** Gene ontology (GO) analysis of the 22 proteins showing a significant difference between immune and pre-immune sera. The analysis was carried out using the STRING database (https://string-db.org (accessed on 7 July 2020)). The focus was placed on “cellular components” and the results have been classified using the “strength” parameter in a descending order.

Cellular Components						
# Term ID	Term Description	Observed Gene Count	Background Gene Count	Strength	False Discovery Rate	Matching Proteins in Your Network (Labels)
GO:0035686	Sperm fibrous sheath	2	12	2.31	0.0037	GAPDHS, AKAP3
GO:0002199	Zona pellucida receptor complex	2	13	2.28	0.0037	CCT3, HSPA1L
GO:0097228	Sperm principal piece	2	29	1.93	0.0094	GAPDHS, AKAP3
GO:0043209	Myelin sheath	6	212	1.54	1.91e-06	CCT3, HSPD1, TUBB4B, PEBP1, GPI1, GAPDH
GO:0005740	Mitochondrial envelope	4	590	0.92	0.0283	HSPD1, CRAT, PEBP1, DIABLO
GO:0031967	Organelle envelope	5	1015	0.78	0.0283	HSPD1, CRAT, PEBP1, DIABLO, GAPDH

## Data Availability

All data are presented in figures, tables and supplementary materials.

## Supplementary Materials

Supplementary Materials can be found at https://www.mdpi.com/article/10.3390/ijms22189965/s1.
