# Supplementary material for: Identification of Arvicola terrestris scherman Sperm Antigens for Immune Contraceptive Purposes
Source: Int J Mol Sci. 2021 Sep 15;22(18):9965. doi: 10.3390/ijms22189965 (PMC8465135; doi:10.3390/ijms22189965)
Supplement: Supplementary file 1 [file ijms-22-09965-s001.zip › ijms-1368787-supplementary.pdf]

Supplementary

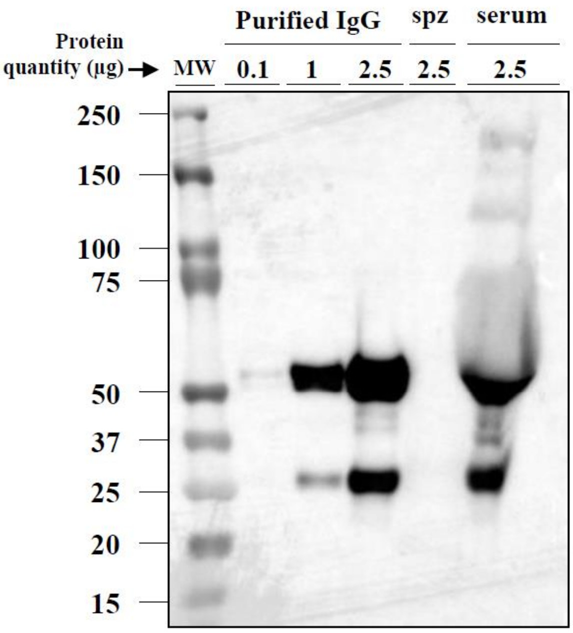

<u>**Figure S1**</u>**: Specificity of the HRP-conjugated secondary antibody directed against** ***ATS*** **IgG.** The reactivity of the generated antibody was verified against purified *ATS* IgG (0.1, 1 and 2.5 μg, left wells), total *ATS* sperm protein extracts (2.5 μg) or proteins from *ATS* serum (2.5 μg, right well) in a 12% SDS-PAGE gel. The secondary anti-IgG antibody recognized the IgG heavy and light chains at 50 and 25 kDa respectively, only in purified IgG from *ATS* and *ATS* serum. These chains are not observed with protein extracts of *ATS* sperm. The HRP-conjugated anti-*ATS* IgG secondary antibody therefore specifically recognized the *ATS* IgG, thus confirming its specificity.

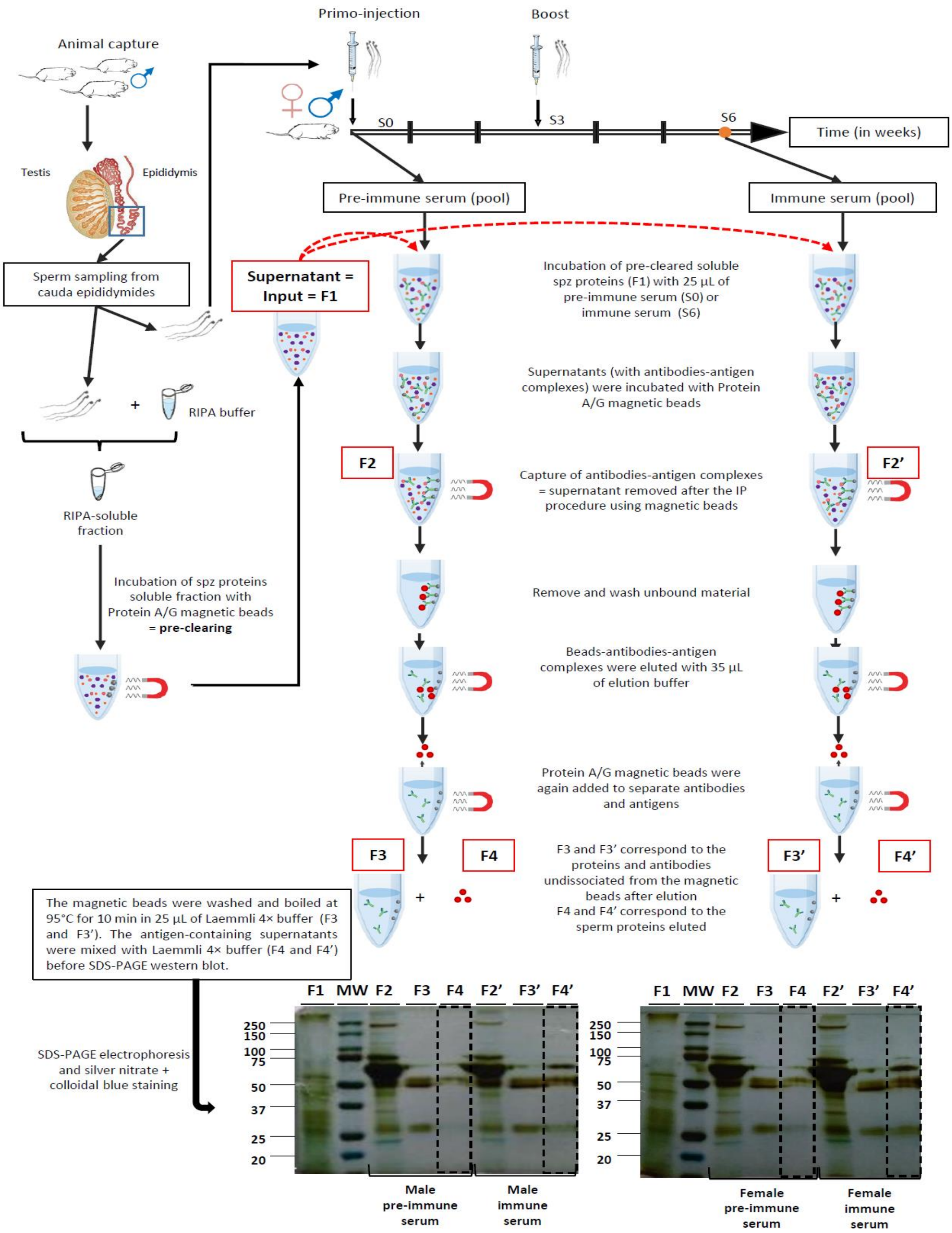

Figure S2: Immunoprecipitation of *ATS* sperm proteins recognized by IgM and IgG type antibodies in male and female sera. The diagram in the upper part of the figure describes the immunoprecipitation strategy. The proteins present in the different fractions mentioned in the diagram are revealed in the lower part of the figure, using a colloidal blue and silver nitrate stained 12% SDS-PAGE gel. Proteins in the F4 and F4′ fractions correspond to the sperm proteins specifically immunoprecipitated by the antibodies and eluted in the final step. Bands at molecular weights of around 75-70, 60-50 and 30-25 kDa were revealed in these fractions with both immune and pre-immune sera, in accordance with the spots found by 2D electrophoresis. The experiment was performed in four replicates with the same sera but with different sperm protein lysates from three

different *ATS*. The proteins contained in the wells framed in black dotted lines (F4 anf F4′) were further analyzed by mass spectrometry.
